# Di-μ-chlorido-bis­{aqua­chlorido[2,2′-thio­bis(pyridine *N*-oxide)-κ*O*]copper(II)}

**DOI:** 10.1107/S1600536809011076

**Published:** 2009-03-28

**Authors:** Rüdiger W. Seidel, Iris M. Oppel

**Affiliations:** a Analytische Chemie, Ruhr-Universität Bochum, Universitätsstrasse 150, 44780 Bochum, Germany

## Abstract

The crystal structure of the title compound, [Cu_2_Cl_4_(C_10_H_8_N_2_O_2_S)_2_(H_2_O)_2_], comprises neutral centrosymmetric μ-chloride-bridged dinuclear units. Each Cu^II^ ion is penta­coordinated by three chloride ligands, a pyridine *N*-oxide O atom and a water mol­ecule. Intra- and inter­molecular O—H⋯O hydrogen bonds occur between the coordinated water mol­ecules and the uncoordinated and coordinated pyridine *N*-oxide groups of the 2,2′-thio­bis(pyridine *N*-oxide) ligands, respectively.

## Related literature

For the potential of pyridine *N*-oxide-based building blocks in the construction of coordination polymers and crystal engin­eering, see: Sun *et al.* (2008 ▶) and references cited therein. For details of hydrogen-bond motifs, see: Bernstein *et al.* (1995 ▶). For a copper-catalysed example of *in situ* S—S and S—C*sp* ^2^ bond cleavage and rearrangement of an related disulfide, see: Wang *et al.* (2007 ▶).
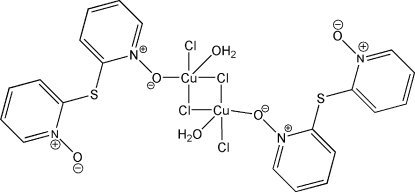


## Experimental

### 

#### Crystal data

[Cu_2_Cl_4_(C_10_H_8_N_2_O_2_S)_2_(H_2_O)_2_]
*M* *_r_* = 745.40
Monoclinic, 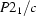

*a* = 6.7552 (18) Å
*b* = 11.430 (3) Å
*c* = 17.375 (3) Å
β = 95.516 (17)°
*V* = 1335.4 (6) Å^3^
*Z* = 2
Mo *K*α radiation
μ = 2.19 mm^−1^
*T* = 294 K
0.27 × 0.21 × 0.19 mm

#### Data collection

Siemens P4 four-circle diffractometer
Absorption correction: ψ scan (*ABSPsiScan* in *PLATON*; Spek, 2009 ▶) *T*_min_ = 0.529, *T*_max_ = 0.663
3316 measured reflections
2349 independent reflections
1736 reflections with *I* > 2(*I*)
*R*_int_ = 0.058
3 standard reflections every 97 reflections intensity decay: none

#### Refinement

*R*[*F* ^2^ > 2σ(*F* ^2^)] = 0.040
*wR*(*F* ^2^) = 0.088
*S* = 1.02
2349 reflections
178 parameters
2 restraints
H atoms treated by a mixture of independent and constrained refinement
Δρ_max_ = 0.40 e Å^−3^
Δρ_min_ = −0.53 e Å^−3^

### 

Data collection: *XSCANS* (Bruker, 1999 ▶); cell refinement: *XSCANS*; data reduction: *XSCANS*; program(s) used to solve structure: *SHELXS97* (Sheldrick, 2008 ▶); program(s) used to refine structure: *SHELXL97* (Sheldrick, 2008 ▶); molecular graphics: *DIAMOND* (Brandenburg, 2008 ▶); software used to prepare material for publication: *SHELXL97*.

## Supplementary Material

Crystal structure: contains datablocks I, global. DOI: 10.1107/S1600536809011076/dn2439sup1.cif

Structure factors: contains datablocks I. DOI: 10.1107/S1600536809011076/dn2439Isup2.hkl

Additional supplementary materials:  crystallographic information; 3D view; checkCIF report

## Figures and Tables

**Table 1:** Hydrogen-bond geometry (Å, °)

*D*—H⋯*A*	*D*—H	H⋯*A*	*D*⋯*A*	*D*—H⋯*A*
O2—H2*A*⋯O1^i^	0.81 (2)	2.13 (2)	2.919 (4)	167 (5)
O2—H2*B*⋯O11^ii^	0.82 (2)	1.97 (2)	2.789 (5)	177 (5)

Symmetry codes: (i) 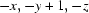; (ii) 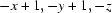.

**Fig. 1.**
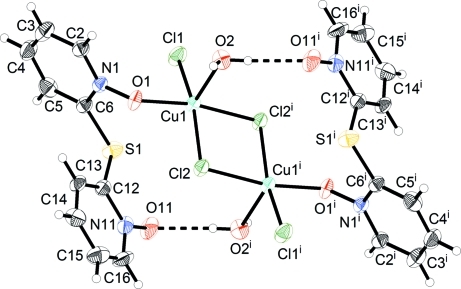
ORTEP diagram of the title compound with 50% probability of the displacement ellipsoids. Hydrogen atoms are drawn at arbitrary size. Hydrogen bonds are represented by dashed lines. For the symmetry codes see Table 1.

**Fig. 2.**
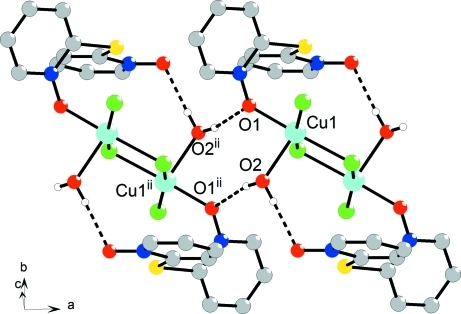
Hydrogen bonding interactions between to adjacent molecules in the crystal structure of the title compound. Hydrogen bonds are represented by dashed lines. For the symmetry codes see Table 2.

## supplementary crystallographic information

### Comment

Pyridine-N-oxide based ligands have attracted a considerable interest in crystal engineering and the synthesis of coordination polymers (Sun *et al.*, 2008).

The title compound, namely bis(µ-chlorido)-diaqua-dichlorido- bis(2,2'-thiobis(pyridine-N-oxide-κ*O*)-dicopper(II), is a neutral dinuclear complex with a central Cu_2_Cl_2_-ring exhibiting *C*_i_ point symmetry (Fig. 1). The unit cell contains two molecules which reside on a crystallographic centre of inversion. Each Cu^2+^ ion adopts a distorted square-pyramidal coordination sphere. Two equatorial *cis*-coordination sites are occupied by the two bridging chlorido ligands. Another chlorido ligand in monodentate coordination mode and an oxygen atom of the pyridine-N-oxide group of the 2,2'-thiobis(pyridine-N-oxide) are located at the remaining two *cis*-sites. A water molecule binds to the axial position. The molecular geometry parameters are within normal ranges. The dihedral angle Cu(µ-Cl)_2_/CuClO(N-oxide) is 17.0 (1)°. The angle between the mean planes of the rings N1—C6 and N11—C16 is 66.4 (1)°.

The coordinated water molecule forms an intramolecular hydrogen bond to O11 of the non-coordinating pyridine-N-oxide group of the 2,2'-thiobis(pyridine-N-oxide) ligand. The graph set here is *S*(12) (Bernstein *et al.*,  1995). The second water hydrogen atom is involved in an intermolecular hydrogen bond to O1 of the coordinating pyridine-N-oxide group with a centrosymmetric *R*^2^_2_(8) motif. This leads to the formation of infitine chains *via* hydrogen bonding extending in the [100] direction with a period corresponding to the crystallographic *a* axis. Hydrogen bonding details are listed in Table 2.

To the best of our knowledge the title compound is the first coordination compound and the first crystal structure comprising 2,2-thiobis(pyridine-N-oxide).

### Experimental

A dark-yellow crystal of the title compound suitable for X-ray diffraction was obtained when equimolar amounts of CuCl_2_ and 2,2'-dithiobis(pyridine-N-oxide) (Aldrich) were dissolved in methanol and the solution was left at ambient temperature. The crystal was found whithin dark-green unidentified material. The origin of the new 2,2'-thiobis(pyridine-N-oxide) ligand is not clear. Either a trace impurity in the starting material or an *in situ* cleavage and rearrangement of S—*S* and *S*—C(*sp*^2^) bonds can be considered. A copper catalysed example of the latter with an related disulfide was reported by Wang *et al.* (2007). As far we can ascertain no synthetic route to 2,2'-thiobis(pyridine-N-oxide) has been reported in the literature.

### Refinement

The crystal structure was refined by full-matrix least-squares refinement on *F*^2^. Anisotropic displacement parameters were introduced for all non-hydrogen atoms. Hydrogen atoms were placed at geometrically positions and refined with the appropriate riding model. The water hydrogen atoms were located in a difference Fourier synthesis and refined with O—H distances of 0.82 (2) Å and *U*_iso_ 1.2 times that of the parent oxygen atom.

### Crystal data

**Table:**

[Cu_2_Cl_4_(C_10_H_8_N_2_O_2_S)_2_(H_2_O)_2_]	*F*(000) = 748
*M**_r_* = 745.40	*D*_x_ = 1.854 Mg m^−^^3^
Monoclinic, *P*2_1_/*c*	Mo *K*α radiation, λ = 0.71073 Å
Hall symbol: -P 2ybc	Cell parameters from 25 reflections
*a* = 6.7552 (18) Å	θ = 5.1–18.0°
*b* = 11.430 (3) Å	µ = 2.19 mm^−^^1^
*c* = 17.375 (3) Å	*T* = 294 K
β = 95.516 (17)°	Prism, dark-yellow
*V* = 1335.4 (6) Å^3^	0.27 × 0.21 × 0.19 mm
*Z* = 2	

### Data collection

**Table:**

Siemens P4 four-circle diffractometer	1736 reflections with *I* > 2(*I*)
Radiation source: fine-focus sealed tube	*R*_int_ = 0.058
graphite	θ_max_ = 25.0°, θ_min_ = 2.1°
ω scans	*h* = −8→1
Absorption correction: ψ scan (ABSPsiScan in *PLATON*; Spek, 2009)	*k* = −1→13
*T*_min_ = 0.529, *T*_max_ = 0.663	*l* = −20→20
3316 measured reflections	3 standard reflections every 97 reflections
2349 independent reflections	intensity decay: none

### Refinement

**Table:**

Refinement on *F*^2^	Primary atom site location: structure-invariant direct methods
Least-squares matrix: full	Secondary atom site location: difference Fourier map
*R*[*F*^2^ > 2σ(*F*^2^)] = 0.040	Hydrogen site location: inferred from neighbouring sites
*wR*(*F*^2^) = 0.088	H atoms treated by a mixture of independent and constrained refinement
*S* = 1.01	*w* = 1/[σ^2^(*F*_o_^2^) + (0.0347*P*)^2^] where *P* = (*F*_o_^2^ + 2*F*_c_^2^)/3
2349 reflections	(Δ/σ)_max_ < 0.001
178 parameters	Δρ_max_ = 0.40 e Å^−^^3^
2 restraints	Δρ_min_ = −0.53 e Å^−^^3^

### Special details

**Table:**

Geometry. All e.s.d.'s (except the e.s.d. in the dihedral angle between two l.s. planes) are estimated using the full covariance matrix. The cell e.s.d.'s are taken into account individually in the estimation of e.s.d.'s in distances, angles and torsion angles; correlations between e.s.d.'s in cell parameters are only used when they are defined by crystal symmetry. An approximate (isotropic) treatment of cell e.s.d.'s is used for estimating e.s.d.'s involving l.s. planes.
Refinement. Refinement of *F*^2^ against ALL reflections. The weighted *R*-factor *wR* and goodness of fit *S* are based on *F*^2^, conventional *R*-factors *R* are based on *F*, with *F* set to zero for negative *F*^2^. The threshold expression of *F*^2^ > σ(*F*^2^) is used only for calculating *R*-factors(gt) *etc*. and is not relevant to the choice of reflections for refinement. *R*-factors based on *F*^2^ are statistically about twice as large as those based on *F*, and *R*- factors based on ALL data will be even larger.

### Fractional atomic coordinates and isotropic or equivalent isotropic displacement parameters (Å^2^)

**Table:**

	*x*	*y*	*z*	*U*_iso_*/*U*_eq_	
Cu1	0.36057 (7)	0.52270 (5)	0.07537 (3)	0.02340 (16)	
Cl1	0.42569 (17)	0.52142 (11)	0.20449 (6)	0.0365 (3)	
Cl2	0.34708 (15)	0.57187 (10)	−0.05629 (6)	0.0273 (3)	
S1	0.42443 (17)	0.79699 (11)	0.11518 (7)	0.0343 (3)	
O1	0.1176 (4)	0.6210 (3)	0.07498 (15)	0.0272 (7)	
O2	0.1784 (5)	0.3623 (3)	0.05848 (18)	0.0325 (8)	
H2A	0.086 (5)	0.372 (4)	0.026 (2)	0.039*	
H2B	0.231 (7)	0.305 (3)	0.040 (2)	0.039*	
N1	0.0804 (5)	0.6857 (3)	0.13620 (19)	0.0250 (8)	
C2	−0.0843 (6)	0.6616 (4)	0.1706 (2)	0.0290 (11)	
H2	−0.1683	0.6013	0.1521	0.035*	
C3	−0.1287 (7)	0.7269 (4)	0.2335 (3)	0.0378 (12)	
H3	−0.2435	0.7108	0.2572	0.045*	
C4	−0.0060 (7)	0.8142 (4)	0.2611 (3)	0.0386 (12)	
H4	−0.0342	0.8569	0.3043	0.046*	
C5	0.1619 (7)	0.8391 (4)	0.2242 (3)	0.0352 (11)	
H5	0.2461	0.8996	0.2423	0.042*	
C6	0.2049 (6)	0.7744 (4)	0.1606 (2)	0.0250 (10)	
O11	0.6518 (4)	0.8360 (3)	0.00176 (19)	0.0412 (9)	
N11	0.4647 (5)	0.8581 (3)	−0.0254 (2)	0.0315 (9)	
C12	0.3202 (7)	0.8442 (4)	0.0239 (2)	0.0276 (11)	
C13	0.1254 (6)	0.8656 (4)	−0.0018 (2)	0.0292 (11)	
H13	0.0267	0.8566	0.0315	0.035*	
C14	0.0761 (7)	0.9005 (4)	−0.0771 (3)	0.0378 (12)	
H14	−0.0560	0.9141	−0.0951	0.045*	
C15	0.2246 (8)	0.9151 (4)	−0.1254 (3)	0.0425 (13)	
H15	0.1930	0.9400	−0.1761	0.051*	
C16	0.4167 (8)	0.8932 (4)	−0.0992 (3)	0.0395 (13)	
H16	0.5161	0.9024	−0.1322	0.047*	

### Atomic displacement parameters (Å^2^)

**Table:**

	*U*^11^	*U*^22^	*U*^33^	*U*^12^	*U*^13^	*U*^23^
Cu1	0.0210 (3)	0.0273 (3)	0.0224 (3)	0.0022 (3)	0.0041 (2)	−0.0021 (2)
Cl1	0.0376 (7)	0.0499 (7)	0.0224 (6)	0.0122 (6)	0.0045 (5)	0.0007 (5)
Cl2	0.0246 (6)	0.0331 (6)	0.0249 (6)	0.0063 (5)	0.0050 (4)	−0.0017 (5)
S1	0.0216 (6)	0.0443 (7)	0.0370 (7)	−0.0053 (6)	0.0028 (5)	0.0023 (6)
O1	0.0223 (16)	0.0342 (17)	0.0250 (16)	0.0056 (14)	0.0018 (13)	−0.0109 (14)
O2	0.0259 (19)	0.0323 (19)	0.038 (2)	−0.0022 (16)	−0.0030 (15)	−0.0023 (16)
N1	0.0210 (19)	0.029 (2)	0.0253 (19)	0.0026 (17)	0.0042 (16)	−0.0004 (17)
C2	0.021 (2)	0.031 (3)	0.036 (3)	−0.001 (2)	0.006 (2)	0.000 (2)
C3	0.029 (3)	0.053 (3)	0.033 (3)	0.006 (3)	0.008 (2)	−0.001 (2)
C4	0.046 (3)	0.042 (3)	0.030 (3)	0.008 (3)	0.011 (2)	−0.006 (2)
C5	0.041 (3)	0.028 (3)	0.037 (3)	−0.005 (2)	−0.001 (2)	−0.005 (2)
C6	0.019 (2)	0.030 (2)	0.027 (2)	0.001 (2)	0.0029 (19)	0.0001 (19)
O11	0.0196 (17)	0.047 (2)	0.059 (2)	−0.0015 (16)	0.0111 (16)	−0.0083 (17)
N11	0.023 (2)	0.026 (2)	0.047 (2)	−0.0057 (17)	0.0112 (18)	−0.0080 (19)
C12	0.028 (3)	0.022 (2)	0.035 (3)	−0.008 (2)	0.011 (2)	−0.007 (2)
C13	0.020 (2)	0.032 (3)	0.037 (3)	−0.002 (2)	0.012 (2)	−0.002 (2)
C14	0.037 (3)	0.037 (3)	0.040 (3)	−0.002 (2)	0.002 (2)	0.007 (2)
C15	0.048 (3)	0.049 (3)	0.030 (3)	−0.006 (3)	0.007 (2)	0.002 (2)
C16	0.049 (3)	0.045 (3)	0.028 (3)	−0.010 (3)	0.020 (2)	−0.004 (2)

### Geometric parameters (Å, °)

**Table:**

Cu1—O1	1.988 (3)	C3—H3	0.9300
Cu1—O2	2.212 (3)	C4—C5	1.386 (6)
Cu1—Cl1	2.2443 (12)	C4—H4	0.9300
Cu1—Cl2^i^	2.3031 (12)	C5—C6	1.384 (6)
Cu1—Cl2	2.3489 (12)	C5—H5	0.9300
Cl2—Cu1^i^	2.3031 (12)	O11—N11	1.331 (5)
S1—C12	1.758 (5)	N11—C16	1.353 (6)
S1—C6	1.764 (4)	N11—C12	1.369 (5)
O1—N1	1.339 (4)	C12—C13	1.370 (6)
O2—H2A	0.81 (2)	C13—C14	1.378 (6)
O2—H2B	0.82 (2)	C13—H13	0.9300
N1—C2	1.341 (5)	C14—C15	1.379 (6)
N1—C6	1.359 (5)	C14—H14	0.9300
C2—C3	1.381 (6)	C15—C16	1.357 (7)
C2—H2	0.9300	C15—H15	0.9300
C3—C4	1.355 (7)	C16—H16	0.9300
			
O1—Cu1—O2	91.10 (12)	C3—C4—H4	120.4
O1—Cu1—Cl1	95.13 (8)	C5—C4—H4	120.4
O2—Cu1—Cl1	100.39 (9)	C6—C5—C4	120.3 (4)
O1—Cu1—Cl2^i^	169.61 (9)	C6—C5—H5	119.9
O2—Cu1—Cl2^i^	93.77 (9)	C4—C5—H5	119.9
Cl1—Cu1—Cl2^i^	93.00 (4)	N1—C6—C5	118.5 (4)
O1—Cu1—Cl2	84.65 (8)	N1—C6—S1	119.4 (3)
O2—Cu1—Cl2	95.75 (9)	C5—C6—S1	121.9 (3)
Cl1—Cu1—Cl2	163.86 (5)	O11—N11—C16	121.7 (4)
Cl2^i^—Cu1—Cl2	85.74 (4)	O11—N11—C12	117.8 (4)
Cu1^i^—Cl2—Cu1	94.26 (4)	C16—N11—C12	120.5 (4)
C12—S1—C6	99.6 (2)	N11—C12—C13	119.7 (4)
N1—O1—Cu1	121.8 (2)	N11—C12—S1	110.7 (3)
Cu1—O2—H2A	111 (4)	C13—C12—S1	129.6 (3)
Cu1—O2—H2B	117 (3)	C12—C13—C14	119.9 (4)
H2A—O2—H2B	100 (5)	C12—C13—H13	120.1
O1—N1—C2	117.9 (4)	C14—C13—H13	120.1
O1—N1—C6	120.1 (3)	C13—C14—C15	119.3 (5)
C2—N1—C6	122.0 (4)	C13—C14—H14	120.3
N1—C2—C3	119.6 (4)	C15—C14—H14	120.3
N1—C2—H2	120.2	C16—C15—C14	120.1 (5)
C3—C2—H2	120.2	C16—C15—H15	120.0
C4—C3—C2	120.4 (4)	C14—C15—H15	120.0
C4—C3—H3	119.8	N11—C16—C15	120.5 (4)
C2—C3—H3	119.8	N11—C16—H16	119.7
C3—C4—C5	119.2 (4)	C15—C16—H16	119.7

Symmetry codes: (i) −*x*+1, −*y*+1, −*z*.

### Hydrogen-bond geometry (Å, °)

**Table:**

*D*—H···*A*	*D*—H	H···*A*	*D*···*A*	*D*—H···*A*
O2—H2A···O1^ii^	0.81 (2)	2.13 (2)	2.919 (4)	167 (5)
O2—H2B···O11^i^	0.82 (2)	1.97 (2)	2.789 (5)	177 (5)

Symmetry codes: (ii) −*x*, −*y*+1, −*z*; (i) −*x*+1, −*y*+1, −*z*.
